# CCL5 and CCR5 Interaction Promotes Cell Motility in Human Osteosarcoma

**DOI:** 10.1371/journal.pone.0035101

**Published:** 2012-04-10

**Authors:** Shih-Wei Wang, Hsing-Hsien Wu, Shih-Chia Liu, Po-Chuan Wang, Wen-Chieh Ou, Wen-Yi Chou, Yung-Shuen Shen, Chih-Hsin Tang

**Affiliations:** 1 Department of Medicine, Mackay Medical College, New Taipei City, Taiwan; 2 Department of Thoracic Surgery, Tainan Municipal Hospital, Tainan, Taiwan; 3 Department of Orthopaedics, Mackay Memorial Hospital, Taipei, Taiwan; 4 Department of Gastroenterology, Mackay Memorial Hospital, Hsinchu, Taiwan; 5 Department of General Surgery, Tainan Municipal Hospital, Tainan, Taiwan; 6 Department of Orthopedic Surgery, Kaohsiung Chang Gung Memorial Hospital Medical Center, Kaohsiung, Taiwan; 7 Holistic Education Center, Mackay Medical College, New Taipei City, Taiwan; 8 Department of Pharmacology, School of Medicine, China Medical University, Taichung, Taiwan; 9 Graduate Institute of Basic Medical Science, China Medical University, Taichung, Taiwan; National Center for Scientific Research Demokritos, Greece

## Abstract

**Background:**

Osteosarcoma is characterized by a high malignant and metastatic potential. CCL5 (previously called RANTES) was originally recognized as a product of activated T cells, and plays a crucial role in the migration and metastasis of human cancer cells. It has been reported that the effect of CCL5 is mediated via CCR receptors. However, the effect of CCL5 on migration activity and integrin expression in human osteosarcoma cells is mostly unknown.

**Methodology/Principal Findings:**

Here we found that CCL5 increased the migration and expression of αvβ3 integrin in human osteosarcoma cells. Stimulation of cells with CCL5 increased CCR5 but not CCR1 and CCR3 expression. CCR5 mAb, inhibitor, and siRNA reduced the CCL5-enhanced the migration and integrin up-regulation of osteosarcoma cells. Activations of MEK, ERK, and NF-κB pathways after CCL5 treatment were demonstrated, and CCL5-induced expression of integrin and migration activity was inhibited by the specific inhibitor and mutant of MEK, ERK, and NF-κB cascades. In addition, over-expression of CCL5 shRNA inhibited the migratory ability and integrin expression in osteosarcoma cells.

**Conclusions/Significance:**

CCL5 and CCR5 interaction acts through MEK, ERK, which in turn activates NF-κB, resulting in the activations of αvβ3 integrin and contributing the migration of human osteosarcoma cells.

## Introduction

Regulated upon Activation Normal T cell Expressed and Secreted (RANTES, CCL5), was originally recognized as a product of activated T cells [1]. Now widely established as an inflammatory chemokine, CCL5 is known to mediate chemotactic activity in T cells, monocytes, dendritic cells, natural killer cells, eosinophils, and basophils [2], [3], [4]. CCL5 is associated with chronic inflammatory diseases such as rheumatoid arthritis, inflammatory bowel disease, and cancer [5], [6]. An association between CCL5 expression and cancer has been reported in melanoma, lung, prostate, and pancreatic cancers [7], [8], [9]. The most striking findings thus far have been with breast cancer [7], [8]. Several investigations have reported that CCL5 was detected in samples from patients with breast cancer and that expression levels correlated with disease progression [7], [8].

Osteosarcoma is a high-grade malignant bone neoplasm that occurs primarily in children and adolescents. The principles of treatment of osteosarcoma have undergone dramatic changes in the past 20 years. Until recently, 5-year survival of 20% with surgical treatment alone was considered acceptable. This outcome suggested that 80% of the patients had pulmonary metastasis at the time of presentation [10]. Hence, chemotherapy is usually employed in an adjuvant situation to improve the prognosis and long-term survival. Recurrence usually occurs as pulmonary metastases or, less frequently, metastases to distant bones or as a local recurrence [11], [12], [13]. Thus, a novel strategy that would efficiently inhibit metastasis, especially to the lung, from the primary osteosarcoma site is highly desirable.

Decades of scrutiny into the molecular bases of cancer have largely focused on what causes oncogenic transformation and the incipient emergence of tumors [14]. The invasion of tumor cells is a complex, multistage process. To facilitate cell motility, invading cells need to change the cell-cell adhesion properties, rearrange the extracellular matrix (ECM) environment, suppress anoikis, and reorganize their cytoskeletons [15]. Integrins are a family of transmembrane adhesion receptors comprising 19 α and 8 β subunits that interact noncovalently to form up to 24 different heterodimeric receptors. The combination of different integrin subunits on the cell surface allows cells to recognize and respond to a variety of different ECM proteins including fibronectin, laminin, collagen, and vitronectin [16]. Because integrins are the primary receptors for cellular adhesion to ECM molecules, they act as crucial transducers of bidirectional cell signaling, regulating cell survival, differentiation, proliferation, migration, and tissue remodeling [17]. Activation and elevated expression of integrin-coupled signaling effectors have been implicated in the induction of a wide variety of human cancers, including those of the breast, colon, prostate, and ovaries [18]. In addition, integrin has also been implicated in metastasis of lung, breast, bladder, and colon cancers [19], [20], [21].

Previous studies have shown that CCL5 modulates cell migration and invasion in human cancer cells [9], [22], [23]. Interaction of CCL5 with its specific receptor CCRs on the surface of cancer cells has been reported to induce cancer invasion [6], [22], [23]. However, the effect of CCL5 and CCR receptor on integrins expression and migration activity in human osteosarcoma cells is mostly unknown. Here we found a phenomenon whereby CCL5 and CCR5 interaction increased the migration and expression of αvβ3 integrin in human osteosarcoma cells. In addition, MAPK kinase (MEK), ERK, and NF-κB signaling pathways were involved.

## Materials and Methods

### Materials

Protein A/G beads, anti-mouse and anti-rabbit IgG-conjugated horseradish peroxidase, rabbit polyclonal antibodies specific for p-MEK, MEK, p-ERK, ERK, p-p65, p65, CCR5 siRNA, control siRNA, control shRNA plasmid, and CCL5 shRNA plasmid were purchased from Santa Cruz Biotechnology (Santa Cruz, CA, USA). Mouse monoclonal antibody specific for αvβ3 integrin was purchased from Chemicon (Temecula, CA, USA). U0126, PD98059, TPCK, and PDTC were purchased from Calbiochem (San Diego, CA, USA). Mouse monoclonal antibody specific for CCR5 and Met-RANTES were purchased from R&D Systems (Minneapolis, MN, USA). The recombinant human CCL5 was purchased from PeproTech (Rocky Hill, NJ, USA). The MEK1 dominant-negative mutant was a gift from Dr. W.M. Fu (National Taiwan University, Taipei, Taiwan). The ERK2 dominant-negative mutant was a gift from Dr. M. Cobb (South-Western Medical Center, Dallas, TX). The NF-κB luciferase plasmid was purchased from Stratagene (La Jolla, CA, USA). pSV-β-galactosidase vector and luciferase assay kit were purchased from Promega (Madison, MA, USA). All other chemicals were purchased from Sigma-Aldrich (St. Louis, MO, USA).

### Cell culture

The human osteosarcoma cell lines (U2OS and MG63) were purchased from the American Type Cell Culture Collection (Manassas, VA, USA). The cells were maintained in RPMI-1640 medium which was supplemented with 20 mM HEPES and 10% heat-inactivated FCS, 2 mM-glutamine, penicillin (100 U/ml) and streptomycin (100 µg/ml) at 37°C with 5% CO_2_.

### Migration and invasion assay

The migration assay was performed using Transwell (Costar, NY; pore size, 8-µm) in 24-well dishes. For invasion assay, filters were precoated with 25 µl Matrigel basement membrane matrix (BD Biosciences, Bedford, MA) for 30 min. The following procedures were the same for both migration and invasion assays [24]. Before performing the migration assay, cells were pretreated for 30 min with different concentrations of inhibitors, including the PD98059, U0126, PDTC, TPCK, or vehicle control (0.1% DMSO). Approximately 2×10^4^ cells in 100 µl of serum-free RPMI-1640 medium were placed in the upper chamber, and 300 µl of the same medium containing CCL5 was placed in the lower chamber. The plates were incubated for 24 h at 37°C in 5% CO_2_, then cells were fixed in methanol for 15 min and stained with 0.05% crystal violet in PBS for 15 min. Cells on the upper side of the filters were removed with cotton-tipped swabs, and the filters were washed with PBS. Cells on the underside of the filters were examined and counted under a microscope. Each clone was plated in triplicate in each experiment, and each experiment was repeated at least three times [25].

### Western blot analysis

The cellular lysates were prepared as described previously [26], [27]. Proteins were resolved on SDS-PAGE and transferred to Immobilon polyvinyldifluoride (PVDF) membranes. The blots were blocked with 4% BSA for 1 h at room temperature and then probed with rabbit anti-human antibodies against MEK, p-MEK, ERK, p-ERK, or p-p65 (1∶1000) for 1 h at room temperature. After three washes, the blots were subsequently incubated with a donkey anti-rabbit peroxidase-conjugated secondary antibody (1∶1000) for 1 h at room temperature. The blots were visualized by enhanced chemiluminescence using Kodak X-OMAT LS film (Eastman Kodak, Rochester, NY). Quantitative data were obtained using a computing densitometer and ImageQuant software (Molecular Dynamics, Sunnyvale, CA).

### Flow cytometric analysis

Human osteosarcoma cells were plated in six-well dishes. The cells were then washed with PBS and detached with trypsin at 37°C. Cells were fixed for 10 min in PBS containing 1% paraformaldehyde. After being rinsed in PBS, the cells were incubated with mouse anti-human antibody against integrin (1∶100) for 1 h at 4°C. Cells were then washed again and incubated with fluorescein isothiocyanate-conjugated goat anti-rabbit secondary IgG (1∶100; Leinco Tec. Inc., St. Louis, MO, USA) for 45 min and analyzed by flow cytometry using FACS Calibur and CellQuest software (BD Biosciences, Lincolin, NE, USA).

### ERK kinase activity assay

ERK kinase activity was assessed by ERK Kinase Activity Assay Kit according to manufacturer's instructions (Sigma-Aldrich, St. Louis, MO, USA). ERK activity kit is based on a solid-phase ELISA that uses a specific synthetic peptide as a substrate for ERK and a polyclonal antibody that recognized the phosphorylated form of the substrate.

### Transfection and reporter gene assay

Human osteosarcoma cells were co-transfected with 0.8 µg κB driven luciferase plasmid, 0.4 µg β-galactosidase expression vector. Cells were grown to 80% confluent in 12 well plates and were transfected on the following day by Lipofectamine 2000 (LF2000; Invitrogen). DNA and LF2000 were premixed for 20 min and then applied to the cells. RPMI-1640 containing 20% FCS was added 4 h later. After 24 h transfection, the cells were then incubated with the indicated agents. After further 24 h's incubation, the media were removed, and cells were washed once with cold PBS. To prepare lysates, 100 µl reporter lysis buffer (Promega, Madison, WI) was added to each well, and cells were scraped from dishes. The supernatant was collected after centrifugation at 11,000 g for 2 min. Aliquots of cell lysates (20 µl) containing equal amounts of protein (20–30 µg) were placed into wells of an opaque black 96-well microplate. An equal volume of luciferase substrate was added to all samples, and luminescence was measured in a microplate luminometer. The value of luciferase activity was normalized to transfection efficiency monitored by the co-transfected β-galactosidase expression vector.

### Quantitative real time PCR

Total RNA was extracted from osteosarcoma cells by using a TRIzol kit (MDBio Inc., Taipei, Taiwan). Two µg of total RNA was reverse transcribed into cDNA by using oligo(dT) primer [28], [29]. The quantitative real time PCR (qPCR) analysis was carried out using Taqman® one-step PCR Master Mix (Applied Biosystems, CA). Two µl of cDNA were added per 25-µl reaction with sequence-specific primers and Taqman® probes. Sequences for all target gene primers and probes were purchased commercially (GAPDH was used as internal control) (Applied Biosystems, CA). qPCR assays were carried out in triplicate on an StepOnePlus sequence detection system. The cycling conditions were 10-min polymerase activation at 95°C followed by 40 cycles at 95°C for 15 s and 60°C for 60 s. The threshold was set above the non-template control background and within the linear phase of target gene amplification to calculate the cycle number at which the transcript was detected (denoted C_T_).

### Establishment of stably transfected cells

CCL5 shRNA or control shRNA plasmids are transfected into cancer cells with Lipofetamine 2000 transfection reagent. Twenty-four hours after transfection, stable transfectants are selected in puromycin (Life Technologies) at a concentration of 10 µg/mL. Thereafter, the selection medium is replaced every 3 days. After 2 weeks of selection in puromycin, clones of resistant cells are isolated.

### Statistics

The values given are means ± S.E.M. The significance of difference between the experimental groups and controls was assessed by Student's *t* test. The difference is significant if the *p* value is <0.05.

## Results

### CCL5 and CCR5 interaction induced migration of osteosarcoma cells

**Figure 1:**
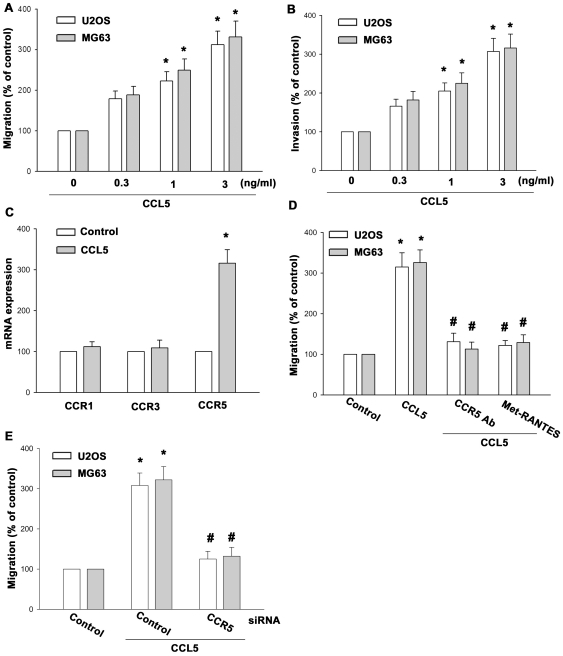
CCL5 and CCR5 interaction increased migration of human osteosarcoma cells. (A&B) MG63 or U20S cells were incubated with various concentrations of CCL5, and *in vitro* migration and invasion activity measured with the Transwell after 24 h. (C) MG63 cells were incubated with CCL5 (3 ng/ml) for 24 h, and the mRNA expression of CCR1, CCR3 and CCR5 was examined by qPCR. MG63 or U20S cells were pretreated for 30 min with CCR5 Ab, Met-RANTES (D) or transfected with CCR5 siRNA (E) for 24 h. Then they were followed by stimulation with CCL5, and *in vitro* migration was measured with the Transwell after 24 h. Results of five independent experiments performed are expressed. Results are expressed as the mean ± S.E. *, p<0.05 compared with control; #, p<0.05 compared with CCL5-treated group.

CCL5 has been reported to stimulate directional migration and invasion of human cancer cells [23], [30]. CCL5-trigered migration in osteosarcoma cells was examined using the Transwell assay. CCL5 directed human osteosarcoma cell migration (U2OS and MG63 cells) (Fig. 1A). We also found that CCL5 increased invasive ability of human osteosarcoma cells through Matrigel basement membrane matrix (Fig. 1B). Interaction of CCL5 with its specific receptor CCR on the surface of cancer cells has been reported to induce cancer migration [7], [22]. Stimulation of cells with CCL5 increased the mRNA expression of CCR5 but not CCR1 and CCR3 (Fig. 1C), suggesting that the amplification loop strengthens the CCL5-CCR5-signaling pathway. Pretreatment of cells with CCR5 mAb or CCR5 receptor inhibitor (Met-RANTES) reduced CCL5-increaed cell migration (Fig. 1D). In addition, transfection of cells with CCR5 siRNA also blocked CCL5-induced cell migration activity (Fig. 1E). Therefore, CCL5 and CCR5 interaction is very important in migration activity of osteosarcoma cells.

### CCL5-directed osteosarcoma cell migration involves αvβ3 integrin up-regulation

**Figure 2:**
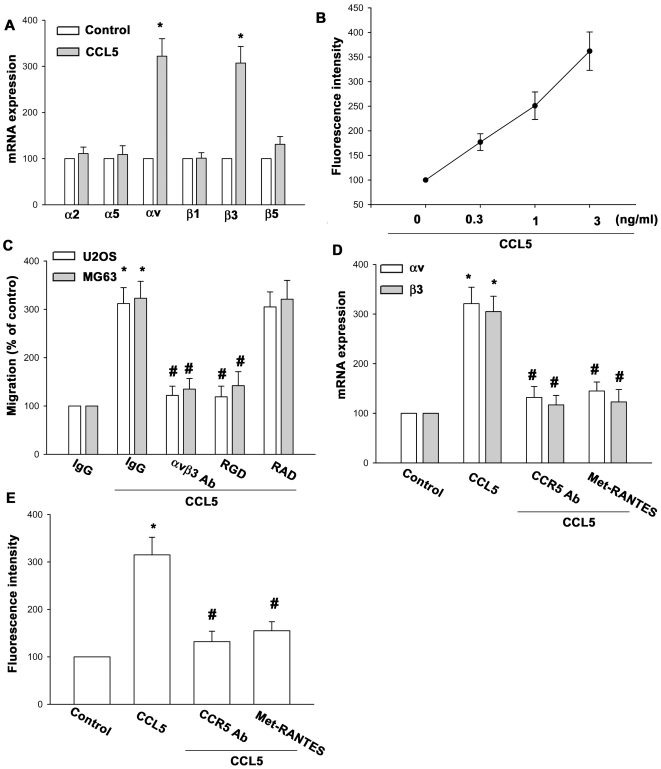
CCL5 and CCR5 interaction induced migration of human osteosarcoma cells involves up-regulation of αvβ3 integrin. (A) MG63 or U20S cells were incubated with CCL5 (3 ng/ml) for 24 h, and the mRNA levels of αv, α2, α5, β1, β3 or β5 integrin was determined using qPCR. (B) MG63 or U20S cells were incubated with CCL5 (0.3–3 ng/ml) for 24 h, and the cell surface expression of αvβ3 integrin was determined using flow cytometry. (C) MG63 or U20S cells were pretreated with αvβ3 monoclonal antibody (10 µg/ml), cyclic RGD (100 nM), or cyclic RAD (100 nM) for 30 min followed by stimulation with CCL5. The *in vitro* migration activity measured after 24 h. (D&E) MG63 or U20S cells were pretreated for 30 min with CCR5 Ab or Met-RANTES followed by stimulation with CCL5. The integrin expression was examined by qPCR and flow cytometry analysis. Results of five independent experiments performed are expressed. Results are expressed as the mean ± S.E. *, p<0.05 compared with control; #, p<0.05 compared with CCL5-treated group.

Previous studies have shown significant expression of integrin in human osteosarcoma cells [31]. We therefore, hypothesized that integrin may be involved in CCL5-directed osteosarcoma cell migration. qPCR analysis showed that CCL5 induced αv and αvβ3 but not α2, α5, β1, and β5 integrin expression (Fig. 2A). To confirm this finding, expression of cell surface integrin in response to CCL5 was analyzed by flow cytometry. Treatment of MG63 cells with CCL5 induced the cell surface expression of αvβ3 integrin dose-dependently (Fig. 2B). Pretreatment of cells for 30 min with anti-αvβ3 monoclonal antibody (mAb) (10 µg/ml) markedly inhibited the CCL5-induced cell migration (Fig. 2C). The cyclic RGD peptide (cyclo-RGDfV) has been reported to bind αvβ3 at high affinity and block its function effectively at low concentrations [32]. Treatment of cells with cyclic RGD, but not cyclic RAD, inhibited the CCL5-induced migration of osteosarcoma (Fig. 2C). Furthermore, pretreatment of cells with CCR5 mAb or Met-RANTES also blocked CCL5-increased αvβ3 integrin expression (Fig. 2D&E). These data suggest that CCL5-induced cancer migration through αvβ3 integrin up-regulation.

### MEK and ERK signaling pathways are involved in the CCL5-mediated cell migration of osteosarcoma cells

**Figure 3:**
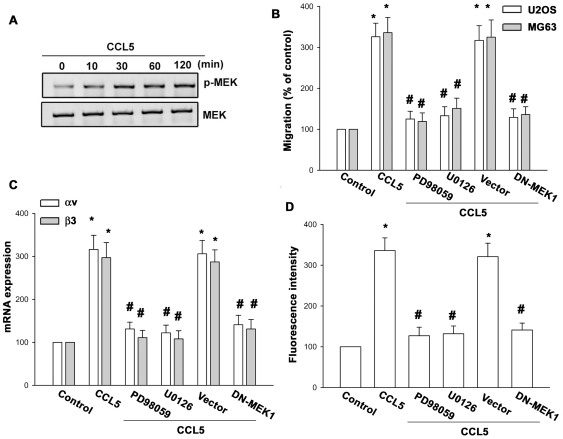
MEK is involved in CCL5-mediated migration in human osteosarcoma cells. (A) MG63 cells were incubated with CCL5 for indicated time intervals, and p-MEK expression was determined by Western blotting. (B–D) MG63 or U20S cells were pretreated with PD98059 and U0126 for 30 min or transfected with dominant negative (DN) mutant of MEK1 for 24 h followed by stimulation with CCL5. The *in vitro* migration and integrin expression was examined by Transwell, qPCR, and flow cytometry analysis. Results of five independent experiments performed are expressed. Results are expressed as the mean ± S.E. *, p<0.05 compared with control; #, p<0.05 compared with CCL5-treated group.

**Figure 4:**
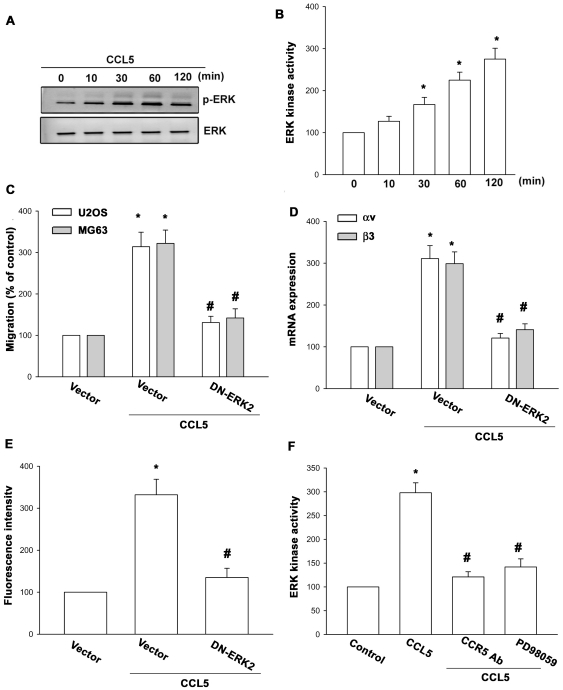
ERK is involved in CCL5-mediated migration in human osteosarcoma cells. (A) MG63 cells were incubated with CCL5 for indicated time intervals, and p-ERK expression was determined by Western blotting. (B) MG63 cells were incubated with CCL5 for indicated time intervals, and ERK kinase activity was examined by ERK kinase assay kit. (C–E) MG63 or U20S cells were transfected with dominant negative (DN) mutant of ERK2 for 24 h followed by stimulation with CCL5. The *in vitro* migration and integrin expression was examined by Transwell, qPCR, and flow cytometry analysis. Results of five independent experiments performed are expressed. Results are expressed as the mean ± S.E. *, p<0.05 compared with control; #, p<0.05 compared with CCL5-treated group.

MEK/ERK signaling pathway can be activated by a variety of growth factors, such as insulin and growth factors [33]. We then examined whether CCL5 stimulation also enhances the activation of the MEK/ERK pathway. Stimulation of cells with CCL5 induced MEK phosphorylation (Fig. 3A). CCL5-induced the migration of osteosarcoma cells were greatly reduced by treatment with MEK inhibitors PD98059 and U0126 (Fig. 3B). The MEK inhibitors PD98095 and U0126 also inhibited the CCL5-increased αvβ3 integrin expression (Fig. 3C&D). Transfection of cells with MEK1 reduced the CCL5-mediated cell migration and αvβ3 integrin expression (Fig. 3B–D). Next, we examined whether ERK involved in CCL5-induced cell migration and integrin expression in human osteosarcoma cells. Stimulation of cells with CCL5 enhanced ERK phosphorylation and kinase activity (Fig. 4A&B). In addition, transfection of cells with ERK2 mutant reduced the CCL5-mediated cell migration and αvβ3 integrin expression (Fig. 4C–E). Furthermore, CCL5-induced ERK kinase activity was markedly inhibited if cells were pretreated for 30 min with CCR5 mAb or PD98059 (Fig. 4F). Taken together, these results indicate that the CCR5, MEK, and ERK pathway is involved in CCL5-induced migration activity and αvβ3 integrin up-regulation in human osteosarcoma cells.

### Involvement of NF-κB in CCL5-induced cell migration and integrin expression

**Figure 5:**
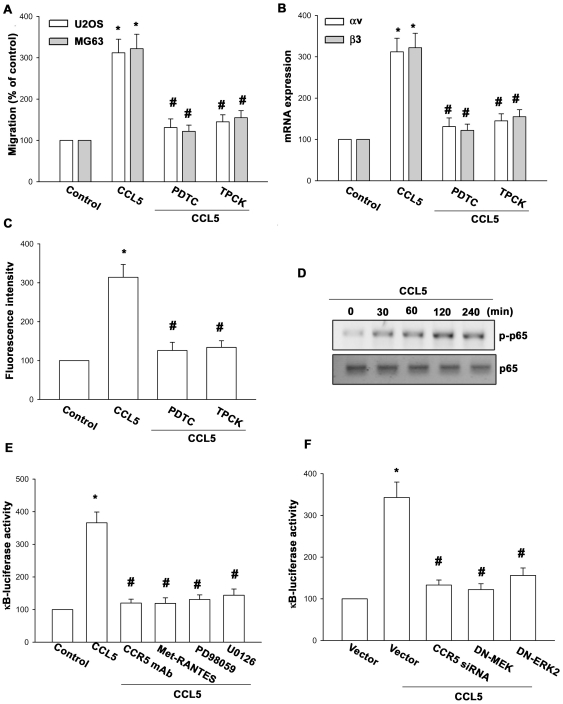
CCL5 induces cell migration and αvβ3 integrin expression through NF-κB. (A–C) MG63 or U20S cells were pretreated for 30 min with PDTC or TPCK followed by stimulation with CCL5. The *in vitro* migration and integrin expression was examined by Transwell, qPCR, and flow cytometry analysis. (D) MG63 cells were incubated with CCL5 for indicated time intervals, and p-p65 expression was determined by Western blotting. (E&F) MG63 cells were pretreated with CCR5 Ab, Met-RANTES, PD98059, and U0126 for 30 min or co-transfected with MEK1 and ERK2 mutant or CCR5 siRNA before exposure to CCL5. The NF-κB driven luciferase activity was measured, and the results were normalized to the β-galactosidase activity and expressed as the mean ± S.E. for three independent experiments performed in triplicate. Results of five independent experiments performed are expressed. Results are expressed as the mean ± S.E *, p<0.05 compared with control; #, p<0.05 compared with CCL5-treated group.

As previously mentioned, NF-κB activation is necessary for the migration and invasion of human osteosarcoma cells [31]. To examine whether NF-κB activation is involved in the signal transduction pathway caused by CCL5 that leads to cell migration and integrin expression, the NF-κB inhibitor pyrrolidine dithiocarbamate (PDTC) was used. Fig. 5A–C show that PDTC inhibited the enhancement of cell migration and αvβ3 integrin expression induced by CCL5. Furthermore, pretreatment of osteosarcoma cells with an IκB protease inhibitor [L-1-tosylamido-2-phenylenylethyl chloromethyl ketone (TPCK)] antagonized the potentiating action of cell migration and αvβ3 integrin expression (Fig. 5A–C). Previous studies showed that p65 Ser^536^ phosphorylation increases NF-κB transactivation [34], and the antibody specific against phosphorylated p65 Ser^536^ was used to examine p65 phosphorylation. Treatment of osteosarcoma cells with CCL5 for various time intervals resulted in p65 Ser^536^ phosphorylation (Fig. 5D). To directly determine NF-κB activation after CCL5 treatment, osteosarcoma cells were transiently transfected with κB-luciferase as an indicator of NF-κB activation. As shown in Fig. 5E, CCL5 treatment of osteosarcoma cells for 24 h caused increase in κB-luciferase activity. In addition, the CCL5-induced increase in κB-luciferase activity was also inhibited by treatment with CCR5 mAb, Met-RANTES, PD98059, and U0126 (Fig. 5E). Co-transfection with MEK and ERK mutant or CCR5 siRNA also reduced CCL5-increased NF-κB luciferase activity (Fig. 5F). Taken together, these data suggest that activation of CCR5 receptor, MEK, and ERK are required for CCL5-induced NF-κB activation in human osteosarcoma cells.

### Decrease cell motility in CCL5-shRNA over-expression clone

**Figure 6:**
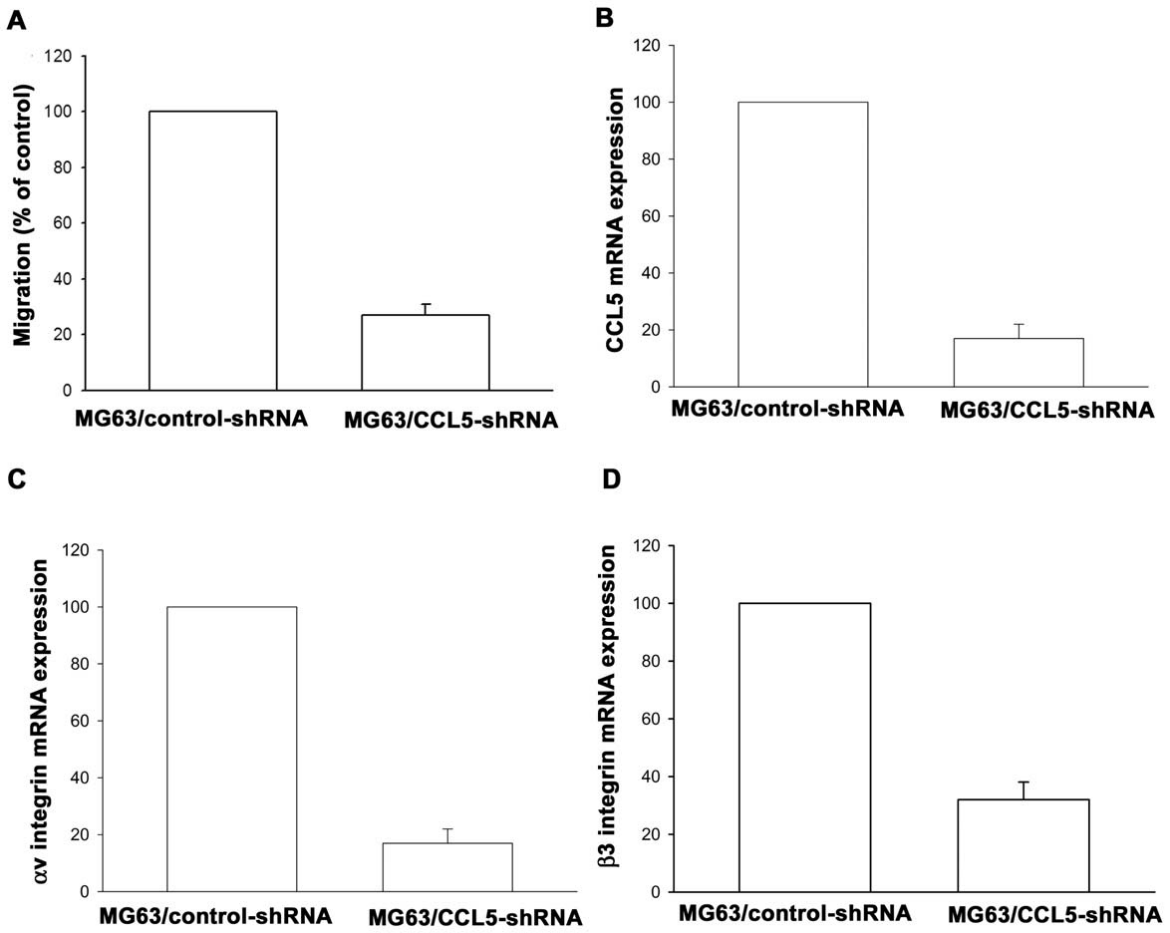
Knockdown of CCL5 inhibited the migratory ability in osteosarcoma cells. (A) The *in vitro* migration activity of MG63/control-shRNA and MG63/CCL5-shRNA cells was measured with the Transwell. (B–D) The mRNA levels of CCL5, αv and β3 integrin in MG63/control-shRNA and MG63/CCL5-shRNA cells was examined by qPCR. Results are expressed as the mean ± S.E.

To further confirm the CCL5 mediated cell migration and integrin expression in human osteosarcoma cells, the CCL5-shRNA expression cells was established. The CCL5 expression level in stable transfectants was compared using qPCR. The expression of CCL5 was dramatically inhibited by CCL5-shRNA orientation in MG63/CCL5-shRNA cells (Fig. 6B). We sought to characterize the cellular growth rate of control cells and transfectants, by performing the MTT assay 1–6 days after cell seeding. No appreciable difference in cell growth ability was evident among these cells (data not shown), suggesting that CCL5 does not have any mitogenic effects in human osteosarcoma cells. Furthermore, the migratory ability of these transfectants was analyzed using a Transwell migration assay. Knockdown of CCL5 expression inhibited the migratory ability by approximately 75% in MG63 cells (Fig. 6A). In addition, knockdown CCL5 also reduced αv and β3 integrin mRNA expression in MG63 cells (Fig. 6C&D). Therefore, knockdown CCL5 reduces cell migration and αvβ3 integrin expression in human osteosarcoma cells.

## Discussion

Osteosarcoma is a debilitating, though not always fatal, high-grade malignant bone neoplasm that targets children and adolescents. The chemotherapies are not fully effective, and as a result, 20% of all patients die due to metastasis of osteosarcomacells to the lungs [12]. Therefore, it is important to develop effective adjuvant therapy for preventing osteosarcoma metastasis. We found that CCL5 increased cell migration in human osteosarcoma cell lines. One of the mechanisms underlying CCL5 directed migration was transcriptional up-regulation of αvβ3 integrin and activation of CCR5 receptor, MEK, ERK, and NF-κB pathways. In previous study has been reported that CCL5 increased lung cancer migration through PI3K/Akt pathway [23]. Chuang et al., reported that PKCδ signaling is mediated CCL5-induced migration in oral cancer cells [22]. Pretreatment of cells with PI3K (Ly294002), Akt (Akt inhibitor), or PKCδ (Rottlerin) inhibitor partially reduced CCL5-induced cell migration in osteosarcoma cells (Figure S1). Therefore, PI3K, Akt, or PKCδ pathways partially involved in CCL5-enhanced cell migration in osteosarcoma. In this study, the only 75% inhibitor of migration after cells treated with MEK, ERK, or NF-κB inhibitor. Combination of PI3K or PKCδ inhibitor with MEK/ERK inhibitor complete reduced CCL5-induced cell migration (Figure S1). Therefore, the other signaling pathways (including PI3K, Akt, and PKCδ) are also partially involved in CCL5-induced cell migration in osteosarcoma cells. On the other hand, we also found that CCL5 promotes the invasion of osteosarcoma cells (Fig. 1B). We therefore examines the matrix metalloproteinases (MMPs) expression after CCL5 stimulation, incubation of cells with CCL5 increased the MMP-2 mRNA expression but not others MMP (Figure S2). Pretreatment of cells with CCR5 mAb, PD98059, U0126, PDTC, or TPCK reduced CCL5-incrased MMP-2 expression (Figure S2). Therefore, the same signaling pathway involved in CCL5-induced cell migration and MMP-2 expression.

It has been reported that CCL5 mediates its biological activities through activation of G protein–coupled receptors, CCR1, CCR3, or CCR5, and binds to glycosaminoglycans [35]. Stimulation of MG63 cells with CCL5 increased the mRNA expression of CCR5 but not CCR1 and CCR3. Therefore, CCR5 is more important than CCR1 and CCR3 in CCL5-mediated cell functions. In this study, we found that both CCR5 and αvβ3 integrin receptor up-regulated after CCL5 stimulation. In addition, CCR5 Ab, Met-RANTES, or CCR5 siRNA also blocked CCL5-incrased αvβ3 integrin expression. Therefore, CCR5 is upstream receptor involved in CCL5-increaed cell migration and integrin expression. Whether other physical or functional connection between these receptors is needs further examination.

Integrins link the ECM to intracellular cytoskeletal structures and signaling molecules and are implicated in the regulation of a number of cellular processes, including adhesion, signaling, motility, survival, gene expression, growth, and differentiation [36]. Here we found that CCL5 increased αvβ3 integrin expression by using flow cytometry analysis, which plays an important role during tumor metastasis. Furthermore, CCL5 also increased the mRNA levels of αv and β3 integrins. In the present study, we used αvβ3 integrin antibody to determine the role of αvβ3 integrin and found that it inhibited CCL5-induced cancer migration, indicating the possible involvement of αvβ3 integrin in CCL5-induced migration in osteosarcoma cells. This was further confirmed by the result that the cyclic RGD inhibited the enhancement of migration activity by CCL5, indicating the involvement of αvβ3 integrin in CCL5-mediated induction of cancer migration.

MEK/ERK pathway plays a critical role in CCL5/CCR5 signaling [37]. We found that PD98059 and U0126 (MEK inhibitors) inhibited CCL5-induced migration. Stimulation of cells with CCL5 increased phosphorylation of MEK and ERK. CCL5 also increased the ERK kinase activity. Transfection of cells with MEK or ERK mutant blocked CCL5-mediated migration and integrin expression. Furthermore, CCL5-induced ERK kinase activity was antagonized by CCR5 mAb and PD98059, indicating that the CCR5 receptor and MEK occur as the upstream molecules involved in CCL5-induced activation of ERK.

A variety of growth factors stimulate cancer metastasis via signal-transduction pathways that converge to activate NF-κB complex of transcription factors [38]. The results of this study show that NF-κB activation contributes to CCL5-induced αvβ3 integrin expression and migration in human osteosarcoma cells, and that the inhibitors of the NF-κB-dependent signaling pathway, including PDTC or TPCK inhibited CCL5-induced αvβ3 integrin expression and cancer migration. Using transient transfection with κB-luciferase as an indicator of NF-κB activity, we also found that CCL5-induced an increase in NF-κB activity. p65 is phosphorylated at Ser^536^ by a variety of kinases through various signaling pathways, and this enhances the p65 transactivation potential. The results of this study showed that CCL5 increased the phosphorylation of p65. On the other hand, CCR5 Ab, Met-RANTES, PD98059, and U0126 reduced CCL5-mediated NF-κB promoter activity. Our data indicated that CCR5, MEK, ERK, and NF-κB pathways might play important role in the expression of αvβ3 integrin and cell migration of human osteosarcoma cells.

Due to the prognosis of patients with osteosarcoma distant metastasis is generally considered as very poor. Thus, preventing human osteosarcoma metastasis is an important issue nowadays. Our study presents that CCL5 and CCR5 interaction increases the expression of αvβ3 integrin via MEK, ERK, p65, and NF-κB-dependent pathway and increasing migration of human osteosarcoma cells. To the best of our knowledge, this study is first time to attempt to examine the migratory activity of CCL5 in human osteosarcoma. On the other hand, we do not have clinical data to show the expression of CCL5 in osteosarcoma and healthy patients. Whether CCL5 acts through same pathways to induce cell motility in clinic should be examined further. Furthermore, the discovery of CCL5-mediated pathway helps us to understand the mechanism of human osteosarcoma metastasis and may help us to develop effective therapy in the future.

## Supporting Information

Figure S1 **PI3K, Akt, and PKCδ pathways are partially involved in CCL5-induced cell migration.** MG63 cells were incubated with Ly294002, Akt inhibitor, Rottlerin, PD98059 plus Ly294002, or PD98058 plus Rottlerin for 30 min followed by stimulation with CCL5. The *in vitro* migration activity measured after 24 h. Results are expressed as the mean ± S.E. *, p<0.05 compared with control; #, p<0.05 compared with CCL5-treated group.
(TIF)

Figure S2 **CCR5, MEK, ERK, and NF-κB pathway is involved in CCL5-induced MMP-2 expression.** (A) MG63 cells were incubated with CCL5 for 24 h, and the mRNA levels of MMPs was determined using qPCR. (B) MG63 cells were pretreated with CCR5 mAb, PD98059, U0126, PDTC, or TPCK for 30 min followed by stimulation with CCL5. The MMP-2 mRNA expression was determined using qPCR. Results are expressed as the mean ± S.E. *, p<0.05 compared with control; #, p<0.05 compared with CCL5-treated group.
(TIF)
