# Supplementary figures and images for: CCL5 and CCR5 Interaction Promotes Cell Motility in Human Osteosarcoma
Source: PLoS One. 2012 Apr 10;7(4):e35101. doi: 10.1371/journal.pone.0035101 (PMC3323659; doi:10.1371/journal.pone.0035101)

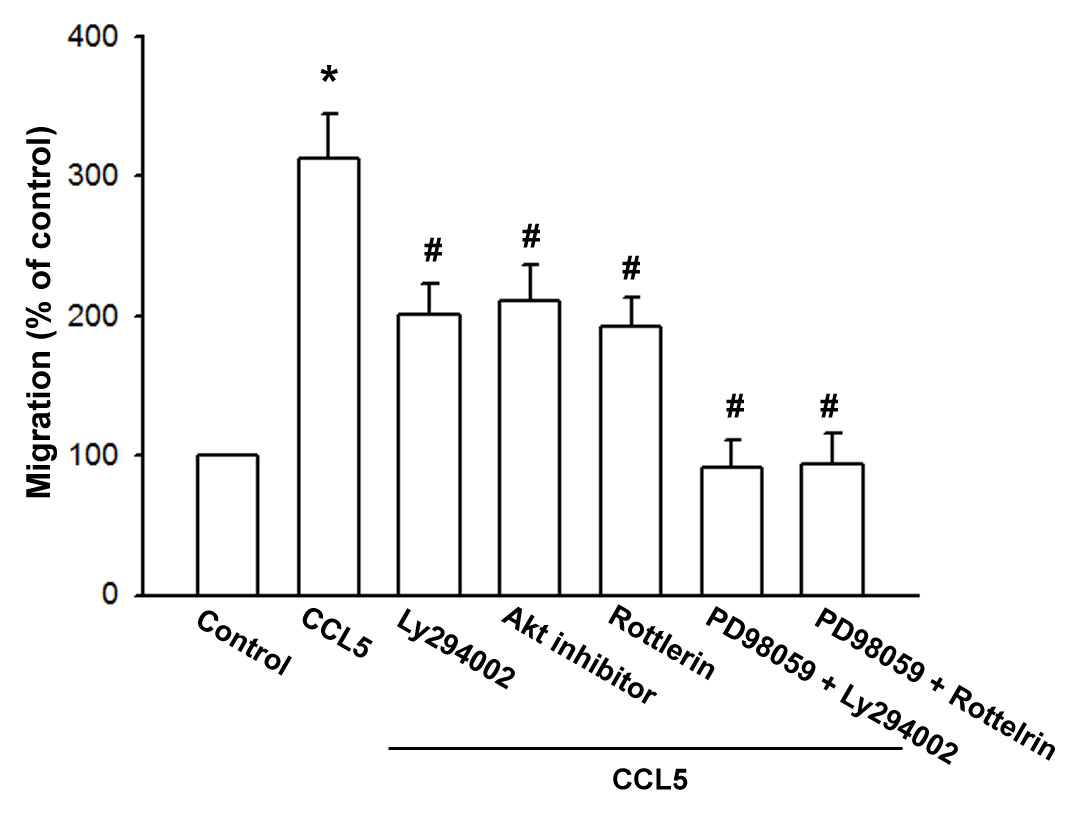

Supplement: Figure S1 — PI3K, Akt, and PKCδ pathways are partially involved in CCL5-induced cell migration. MG63 cells were incubated with Ly294002, Akt inhibitor, Rottlerin, PD98059 plus Ly294002, or PD98058 plus Rottlerin for 30 min followed by stimulation with CCL5. The in vitro migration activity measured after 24 h. Results are expressed as the mean ± S.E. *, p<0.05 compared with control; #, p<0.05 compared with CCL5-treated group. (TIF) [file pone.0035101.s001.tif]

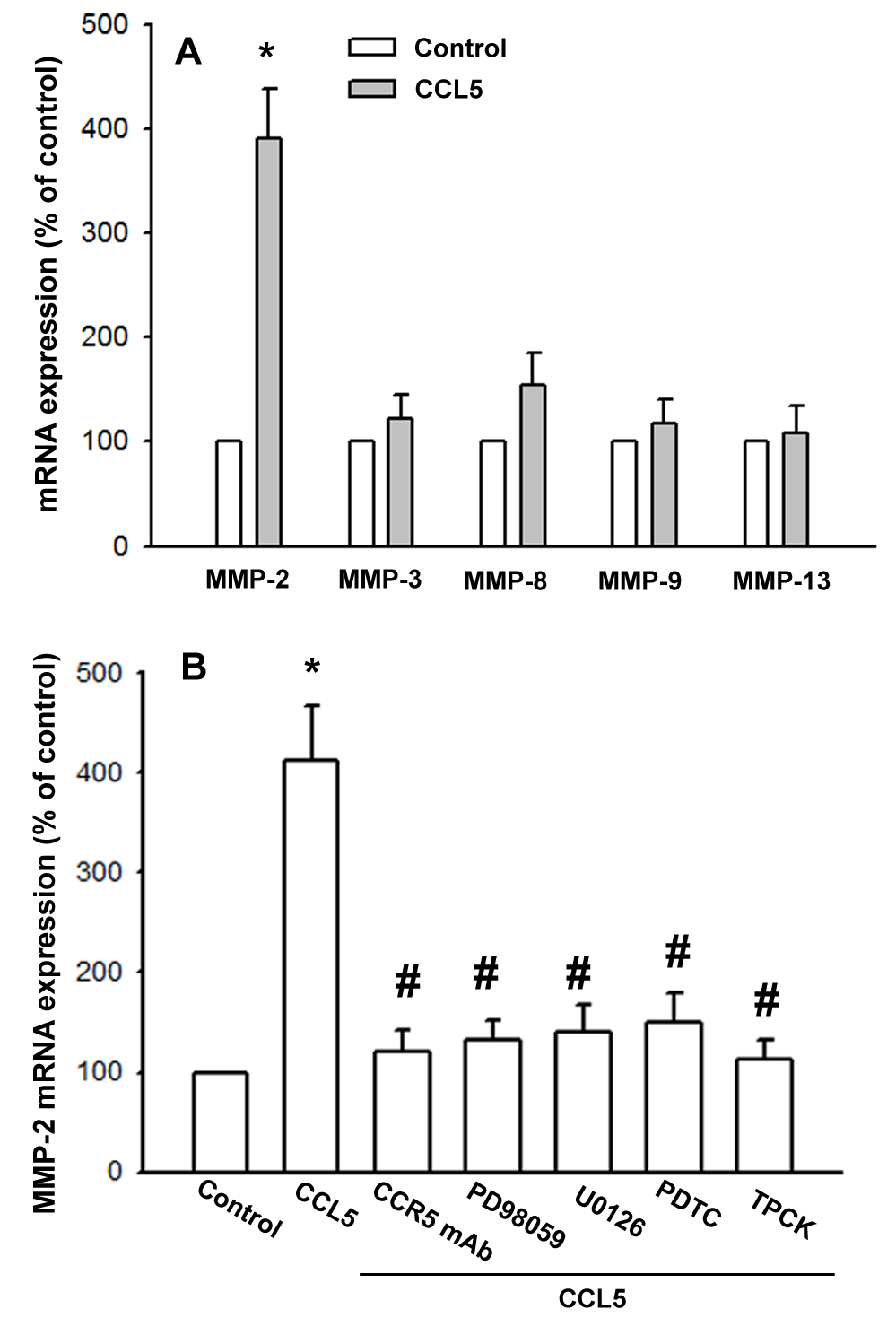

Supplement: Figure S2 — CCR5, MEK, ERK, and NF-κB pathway is involved in CCL5-induced MMP-2 expression. (A) MG63 cells were incubated with CCL5 for 24 h, and the mRNA levels of MMPs was determined using qPCR. (B) MG63 cells were pretreated with CCR5 mAb, PD98059, U0126, PDTC, or TPCK for 30 min followed by stimulation with CCL5. The MMP-2 mRNA expression was determined using qPCR. Results are expressed as the mean ± S.E. *, p<0.05 compared with control; #, p<0.05 compared with CCL5-treated group. (TIF) [file pone.0035101.s002.tif]
